# Taking the Perspective that a Depressive State Reflects Inflammation: Implications for the Use of Antidepressants

**DOI:** 10.3389/fpsyg.2012.00297

**Published:** 2012-08-17

**Authors:** Jill Leslie Littrell

**Affiliations:** ^1^School of Social Work, Georgia State UniversityAtlanta, GA, USA

**Keywords:** major depression, inflammation, antidepressants efficacy, antidepressants, neurogenesis and inflammation

## Abstract

This paper reviews both the evidence that supports the characterization of depression as an inflammatory disorder and the different biochemical mechanisms that have been postulated for the connection between inflammation and depression. This association offers credible explanation for the short term efficacy of antidepressants, which have short term anti-inflammatory effects. Evidence for those anti-inflammatory effects is discussed. Evidence of the contrary long-term effects of antidepressants, which increase rather than decrease inflammation, is also reviewed. It is argued that this increase in inflammation would predict an increase in chronicity among depressed patients that have been treated with antidepressants drugs, which has been noted in the literature. A brief discussion of alternatives for decreasing inflammation, some of which have demonstrated efficacy in ameliorating depression, is presented.

## Depressive Behaviors Involve Inflammation

The association between stress, systemic inflammation, and behavioral signs of depression has been recognized for over a decade (Raison et al., 2006; Capuron et al., 2008; Dantzer et al., 2011). Depressed and anxious persons display elevations in blood levels of inflammatory cytokines [Interleukin-6 (IL-6) and tumor necrosis factor-α (TNF-α)] as well as other markers of an inflammatory state [e.g., C-reactive protein (CRP), soluble intracellular adhesion molecule-1 (ICAM-1), and macrophage chemoattractant protein-1; Rajagopalan et al., 2001; Zorrilla et al., 2001; Schiepers et al., 2005; Raison et al., 2006; Howren et al., 2009; Dowlati et al., 2010], and given a social stressor, depressed individuals exhibit greater elevations in inflammatory cytokines, such as IL-6 (Pace et al., 2006). Depressed persons also exhibit lower levels of anti-inflammatory cytokines (Li et al., 2011).

In addition to the findings for persons exhibiting behavioral signs of depression, the immune system function of individuals undergoing stress has been evaluated. In examining the leukocytes from individuals who reported loneliness, Cole et al. (2007, 2011) found increased transcriptional activity of Nuclear Factor-κB (NF-κB), a marker of inflammatory activation. The spouses of Alzheimer patients and caregivers for cancer patients are characterized by high levels of inflammatory cytokines such as IL-6 (Kiecolt-Glaser et al., 2003; von Kanel et al., 2006) and higher levels of NF-κB activity (Miller et al., 2008). Persons who are under stress at work exhibit elevations in ICAM-1, inflammatory cytokines, and acute phase proteins such as fibrinogen (von Kanel et al., 2001; Steptoe et al., 2003; Ramachandruni et al., 2004; Hong et al., 2006).

### Investigating causal connections

Relatively early in these investigations, Maier and Watkins (1998) advanced the hypothesis that undergoing psychological stress or provoking inflammation in the periphery induces an equivalent state of systemic inflammation. According to Maier and Watkins, systemic inflammation correlates with changes in the brain. Regardless of the initiating event, whether an infection or a psychological stressor, behavioral manifestations of depression emerge that are driven by synonymous changes in the brain. Maier and Watkins speculated that during evolution, an immune system for fighting pathogens emerged first. When multi-celled organisms needed a mechanism for responding to an external psychological threat, the body recruited the already existing immune system for responding to external threat. Similar ideas were proposed by Maes et al. (1999). With regard to the evolutionary connection between psychological stressors and inflammatory activation, Pace et al. (2012) have suggested that given a psychological threat, such as attack by a predator, preparing the immune system to fight infection resulting from a predatory attack could be adaptive.

#### Manipulating psychological stress

Given the association between subjective distress and markers of systematic inflammation, researchers investigated the direction of a possible causal relationship. Learned helplessness is an animal model of depression. To induce learned helplessness, researchers subject animals to bouts of uncontrollable shock. The animal cannot escape or avoid the shock. Subsequent to this stressor, the animal appears depressed. The animal fails to drink sucrose, displays less locomotion, fails to explore new environments, and, even when subsequently given an opportunity to avoid an aversive stimulus, fails to escape or avoid. Animals subjected to helplessness inductions also have elevated levels of IL-1, in their hippocampus and hypothalamus (Maier and Watkins, 1998). Moreover, when an antagonist for the IL-1 is placed in the hippocampi of animals exposed to uncontrollable shock, the animal no longer exhibits depressed behavior, and no longer fails to escape aversive stimuli (Maier and Watkins, 1995; Johnson et al., 2004; Koo and Duman, 2008; Arakawa et al., 2009). Thus, IL-1 in the hippocampus seems to play a causal role in mediating behavioral manifestations of depression.

Another way to make an animal exhibit signs of depression is to expose the animal to repeated episodes of social defeat. After exposure to rounds of social defeat the animal avoids other animals, displays reduced preference for sucrose, and appears more depressed on tests such as the forced swim test and the tail suspension test. Concomitant with behavioral signs of depression, animals display elevation in inflammatory cytokines in plasma (Merlot et al., 2004; Kinsey et al., 2007, 2008; Avitsur et al., 2009).

In addition to the work with animals showing that stressing the animal will induce signs of systemic inflammation, there is also experimental work in humans. Persons who are subjected to laboratory stressors exhibit activation of NF-κB (Bierhaus et al., 2003) as well as an increase in inflammatory cytokines such as IL-6 in plasma (Pace et al., 2006). In those individuals who are low on chronic stress, increases in NF-κB activity are correlated with perceived intensity of the laboratory stressor (Wolf et al., 2009). A study by Slavich et al. (2010) examined how a social stressors inducing elevations in inflammatory cytokines changes brain function. Following the social stressor, those with elevations in the soluble receptor for TNF-α exhibited greater activation in the dorsal anterior cingulate and the anterior insula, two areas involved in processing negative affect.

#### Manipulating peripheral inflammation

Studies established that stressing the animal in various ways results in behavioral manifestations of depression along with elevations in inflammatory cytokines in brain and in plasma. The next question was whether establishing systemic inflammation in the periphery would result in behavioral manifestations of depression. Placing lipopolysaccharide (LPS) molecules in the paw of a mouse is a reliable way to elevate cytokines in the blood stream without inducing real tissue damage to the organism from the bacteria. Following elevations in cytokines in the blood stream, the mice exhibit a lack of preference for formerly preferred foods, low levels of activity, more social avoidance, less exploration of novel environments, an increase in body temperature, and an increase in the production of cortisol (Dantzer, 2001; Nadjar et al., 2005). Concomitant with the behavioral change, the following are observed: an increase in the threshold for self-stimulation in the medial forebrain bundle (Borowski et al., 1998), a diminution in activity in brain areas associated with positively motivated behavior (Stone et al., 2007), an increase in activation as detected by elevations in FosB/ΔFosB in areas of brain associated with depression (viz., parts of the shell of the Nucleus Accumbens, bed nucleus of the stria terminalis (BNST), and the central nucleus of the amygdala; Frenois et al., 2007; Engler et al., 2011). Moreover, IL-1 levels in brain (the hippocampus and hypothalamus) are elevated. Again, when IL-1 antagonists are placed into the brain, the depressed behaviors disappear, although it is noted that timing for the administration of the IL-1 receptor antagonist (IL-1ra) and placement of the IL-1ra can alter the parameters of the response to LPS that are abrogated (Bluthe et al., 1995; Konsman et al., 2008).

In addition to the animal work, similar work in humans is available. Several of these studies observed random-assignment to either peripheral induction of systemic inflammation or to a control condition. Those who received endotoxin administration displayed impairment in memory which correlated with inflammatory cytokine levels (Reichenberg et al., 2001; Krabbe et al., 2005) and an elevation in negative mood which correlated with cytokine levels (Reichenberg et al., 2001). Vaccinating people with typhoid proteins is another reliable way to elevate inflammatory cytokines. Vaccinated individuals displayed elevations in negative mood which correlate with IL-6 levels (Wright et al., 2005).

#### Peripheral inflammation does impact the brain

Since the brain is supposed to be impervious to the entry of cytokines, the question of how cytokines in the periphery communicate with brain has been a subject of inquiry. Several mechanisms have emerged. First, the sensory vagus nerve, which has receptors for IL-1 and prostaglandins (Ek et al., 1998), responds to local inflammation and results in activation in the Nucleus Tractus Solitarius of the medulla which relays information to its projection areas (the parabrachial, paraventricular and supraoptic nuclei and ventromedial preoptic area of the hypothalamus, the central amygdala, and the BNST; Dantzer et al., 2000, 2007; Goehler et al., 2000; Konsman et al., 2000). Second, activated leukocytes in circulation can communicate with brain tissue at areas where the blood brain barrier is weaker: the choroid plexus and circumventricular organs, such as the median eminence, the organum vasculosum lamina terminalis, the subfornical organ, and the area postrema. In these areas, the junctions between the endothelial cells lining blood vessels are less tight and allow for diffusion of cytokines (Konsman et al., 1999; Dantzer et al., 2000). Moreover, inflammatory proteins in plasma, such as monocyte chemoattractive protein-1 (MCP-1), can weaken the blood brain barrier (Stamatovic et al., 2005). Finally, there are active transport molecules on endothelial cells that allow for cytokines to be transported across the blood brain barrier (Dantzer et al., 2007; Shelton and Miller, 2010). Once in the CNS, the cytokines activate the microglia. When microglia get activated, they release MCP-1 which will bring other white blood cells to the brain (Shelton and Miller, 2010).

#### Forces that can restrain systemic inflammation

The body does have mechanisms for curtailing an inflammatory response following clearance of a pathogen. Following activation in response to a pathogen, T regulatory cells (Tregs) are also differentiated. Tregs are identified by their surface expression of CD25 and the expression of the transcription factor: FoxP3. Tregs release IL-10 and transforming growth factor-β (TGF-β), which are anti-inflammatory cytokines (Murphy, 2012).

Data supporting the hypothesis that stress will increase the level of inflammatory cytokines has been presented. It is also the case that a laboratory stressor will decrease the level of IL-10 (Buske-Kirschbaum et al., 2007) as well as the number of circulating Tregs (Freier et al., 2010). Lower levels of Tregs are also observed in those who are chronically stressed as well. Stress will decrease the number of circulating Tregs; will downregulate FoxP3, the transcription factor for Treg differentiation; and decrease plasma levels of IL-10 and TGF-β (Raison et al., 2010c).

A protective role for IL-10 in preventing distress has been established. Administration of IL-10 along with administration of LPS prevents the emergence of behavioral signs of depression (Bluthe et al., 1999). Mice have been genetically engineered to over-express IL-10 display less anxiety, while IL-10 knock outs display greater levels of anxiety and depressive behavior on a forced swim test, although the impact of IL-10 is more pronounced in females (Mesquita et al., 2008). In the human literature, under stressful conditions, those persons with higher levels of IL-10 report less anxiety (Maes et al., 1998). Following vaccination with an inflammatory antigen, optimistic persons maintain higher levels of IL-10 (Costanzo et al., 2004).

Others have attempted to increase T regulatory cells by vaccinating with antigens known to elicit a Tregs response. Vaccination with a heat killed preparation of *Mycobacterium vaccae* will evoke a Treg response (Rook and Lowry, 2008). Those who were vaccinated with *M. vaccae* report better quality of life (Dalbeth et al., 2004; O’Brien et al., 2004).

### Other consistent findings

Beyond the random-assignment controlled research, there are other studies consistent with the hypothesis that systemic inflammation is associated with depressive behaviors. Particular diseases (metastatic melanoma and hepatitis virus C) are treated with Interferon-α (IFN-α). IFN-α will induce systemic inflammation. A significant number of persons (30–50%) undergoing such treatment subsequently develop clinical depression (Raison et al., 2009). In fact, in those being treated with IFN-α, feelings of guilt and suicidal ideation are increased, not just malaise and fatigue (Capuron et al., 2009).

In addition to the data on behavioral impact of IFN-α, several conditions are associated with a rise in inflammatory cytokines. Both obesity and sleep deprivation are associated with elevations in blood levels of inflammatory cytokines (IL-1β, TNF-α). Both these conditions are risk factors for depression (Irwin et al., 2006; Shelton and Miller, 2011). Metabolic syndrome, a state of increased weight around the waist, is associated with high levels of IL-6 and CRP and is also a risk factor for depression (Capuron et al., 2008).

Some of the human research has examined how systemic inflammation alters brain function as observed through functional magnetic resonance imaging. Administration of IFN-α induces activation of the dorsal anterior cingulate-error detection area (Capuron et al., 2005). Inoculation with typhoid proteins, which increases systemic levels of IL-6, similarly enhances activity of the insula and right anterior cingulate which correlate with subjective fatigue and confusion (Harrison et al., 2009). Moreover, inoculation with typhoid proteins results in diminished control of the subgenual anterior cingulate cortex by regulatory centers for reward, emotion, and social processing, a phenomenon which is correlated with levels of IL-6 (Harrison et al., 2009). In addition to inducing changes in areas associated with error processing such as the anterior cingulate cortex, systemic inflammation is correlated with changes in dopaminergic system, the system which undergirds motoric activity and motivation. Following typhoid protein inoculation, levels of IL-6 correlate with altered activity in the substantia nigra and behavioral measures of slower reaction times (Brydon et al., 2008). A study by Eisenberger et al. (2011) also noted changes in dopaminergic structures. LPS administration in the periphery results in less activity in the ventral striatal area (the brain’s reward area) in response to monetary reward.

### Inflammatory cytokines may explain dexamethasone non-suppression

Another consistent finding in the literature is that the level of cortisol hormone is often elevated in depressed individuals, although 30–50% of depressed individuals may not display elevations in cortisol (Muller and Holsboer, 2006; Pace and Miller, 2009). Corticotropin releasing hormone (CRH) from the parvocelluar neurons in the paraventricular nucleus of the hypothalamus is the proximal cause of elevations in cortisol. With regard to regulation of this system, given normal functioning, cortisol will act on receptors for cortisol in the hippocampus. The hippocampus, through connections in the BNST, will then communicate with the paraventricular nucleus of the hypothalamus to inhibit the CRH release. Moreover, the BNST is probably more important in regulating CRH release in situations of chronic stress (Sapolsky, 1985; Ulrich-Lai and Herman, 2009). This is the negative-feedback system for dampening a response. But in depressed individuals, this inhibitory system does not work well. The problem with CRH not being suppressible in depressed individuals may be related to inflammatory states. It has been demonstrated in fibroblast cells that inflammatory cytokines will interfere with glucocorticoid receptors function, although possible variation in cell types is acknowledged (Pariante et al., 1999). Thus, inflammation may be the mechanism creating resistance to control of the cortisol system in depressed individuals (Pariante et al., 1999; Pace et al., 2007). Not only will inflammatory cytokines interfere with regulation of cortisol release, but inflammatory cytokines are also associated with less circadian variation in cortisol release (Raison et al., 2010a).

### Genetic risk

Vulnerability to depression under stressful conditions is conferred by various alleles associated with proteins of the immune system. The short promoter in the gene for the serotonin transporter has received a great deal of attention as a risk factor for depression (Lasky-Su et al., 2005). Interestingly, those carrying the short promoter version of the serotonin transporter release more IL-6 relative to IL-10 given exposure to a laboratory stressor (Fredericks et al., 2010). The less frequently expressed alleles for the serotonin transporter are at increased risk for exhibiting depression given exposure to IFN-α (Bull et al., 2009; Lotrich et al., 2009), although the serotonin transporter gene interacted with alleles for IL-6 only making a difference in those who carried the allele for the less easily expressed IL-6 gene in the Bull et al. study. Particular alleles for TNF-α and IL-1 are also associated with risk for depression or severity of symptoms (Jun et al., 2003; Yu et al., 2003; Raison et al., 2006; Traks et al., 2008). Thus, in looking for genetic vulnerability to major depression, proteins of the immune system may be as or more predictive than proteins associated with neurotransmitter function.

### Questions remain

Questions about the hypothesis that inflammation has a critical role in depression have been raised. The symptoms induced by cytokines versus a psychological stressor may only partially overlap. Clinically, Capuron et al. (2009) compared the phenomenon emerging in those who are treated with IFN-α with the symptomatology in those who are depressed for other reasons. Psychomotor retardation and weight loss predominated in the former whereas guilt was more predominant in the latter. In those treated with IFN-α, sickness behavior emerged immediately whereas depressive symptoms emerged a month after treatment initiation (Capuron and Miller, 2011). In terms of malleability, some interventions may differentially affect sickness behaviors versus depressive behaviors. For example, O’Connor et al. (2009a) administered an inhibitor of indolamine 2,3 dioxygenase (IDO to be discussed later) along with inflammatory cytokines. The IDO inhibitor precluded depressive behaviors (more immobility on a forced swim test and tail suspension test) induced by cytokines while failing to affect locomotor activity, weight change, or fever.

Subtle variations in the response to psychological stressors versus systemic inflammation induced by infection are possible. Immunological symptoms emerge in stages after exposure to a stressor (Maier and Watkins, 2010). The response to cytokines can vary as a function of dosage and will also emerge in stages (Dantzer, 2004; Capuron and Miller, 2011). Moreover, there may be subtypes of depression in which inflammation plays a lesser role. However, the overlap between response to a psychological stressor and response to a pathogen is well established.

### Stress and suppression of some compartments of the immune system

The finding that depressed behaviors are associated with indicators of systemic inflammation was surprising. During the late 1980s and 1990s, the field of psychoneuroimmunology was rapidly developing. Animal work and human studies showed that stress depresses many compartments of immune system function. Seminal observations included the following: Epstein–Barr virus is more likely to be reactivated in medical students during final exams (Glaser et al., 1994). Wound healing is slower in dental students during final exams and in Alzheimer patient caregivers (Kiecolt-Glaser et al., 1995; Marucha et al., 1998). Stressed individuals, such as caregivers of Alzheimer Disease patients, fail to develop a robust antibody response to vaccination (Kiecolt-Glaser et al., 1996; Glaser et al., 2000). Later, the findings that systemic inflammation characterized depressed and stressed individuals emerged. The idea that the body’s response to stress involved an immune activation as well as suppression of some compartments of the immune system seemed contradictory.

The immune system is complex and capable of making many types of responses. A big division of the compartments of the immune system is innate versus adaptive immunity. Innate immunity includes those leukocytes that respond promiscuously, activating in response to many pathogens and other foreign molecules. Many of the inflammatory cytokines are products of the innate system. In contrast, the adaptive immune system refers to those leukocytes (T and B lymphocytes) that respond to only one type of molecule and whose responding takes a period of days to activate. Adaptive immunity is evoked with vaccination. A consensus is beginning to emerge regarding the differential impact of stress on the innate versus the adaptive immune systems. Stress reliably decreases the adaptive immune system as well as decreasing natural killer cell activity. In contrast to the adaptive system, stress activates the innate system, i.e., monocytes and macrophages which release inflammatory cytokines (Maier and Watkins, 1998; Zorrilla et al., 2001; Raison et al., 2006).

Blume et al. (2011) observed that few studies have measured both immune system activation (as evidenced by inflammatory cytokines) and immune system suppression (less proliferation of T and B cells, attenuated Natural Killer Cell Cytotoxicity, lower levels of wound healing) in the same study. However, there is evidence that chronic activity of the innate immune system is associated with less robust response from cells of the adaptive immune system (Maier and Watkins, 1998; Blume et al., 2011). Indeed, exposure of T cells to TNF-α, an inflammatory cytokine, will decrease T cell proliferation and production of cytokines (Lee et al., 2008).

One of the early investigations in the literature on psychoneuroimmunology addressed the question of whether persons undergoing severe stress are more likely to “catch a cold.” The studies showed that persons undergoing major stressors produced more mucous and exhibited more symptoms after inoculation with a flu virus (Cohen, 2005). However, the question of whether more symptoms are reflective of greater activation or lesser activation of the immune system is complicated. Cold symptoms (mucous production, inflamed nasal passages) are phenomena reflecting innate immune system activation. A recent study by Huang et al. (2011) provided further clarification on which arms of the immune system are activated or suppressed in those manifesting symptoms in response to a cold virus. Huang et al., as in the earlier Cohen studies, inoculated research-participants with live influenza and then tracked research-participant responses over time. Those remaining asymptomatic had downregulated the NF-κB pathway of the innate system. However, with regard to the adaptive immune system, there was suggestion of a strong Th1 response to the virus in those who remained asymptomatic. The Huang et al. study thus demonstrated that activation of the innate immune system can accompany a less vigorous response from the adaptive system.

## The Importance of Cytokines in Brain Mediating Behavioral Changes

Researchers have abrogated the behavioral changes in response to LPS in the periphery or learned helplessness induction by placing an antagonist to IL-1β into brain (Maier and Watkins, 1995; Johnson et al., 2004; Koo and Duman, 2008; Arakawa et al., 2009). These findings imply that IL-1β, an inflammatory cytokine, induces changes which eventually result in behavioral manifestations of depression. Other researchers have investigated the downstream changes associated with an increase in IL-1β that might operate as more proximal factors in driving the behavioral manifestations of depressed behavior. Discussion of downstream events subsequent to an increase in IL-1βilluminates the significance of brain derived neurotrophic factor (BDNF), a protein which has been featured in explanations of the mechanisms behind the efficacy of antidepressants, as well as the significance of IDO, which has been purported to link to the serotonergic system.

Immunologists view inflammation as a mechanism for fighting pathogens. Most immunology texts view the primary job of leukocytes as providing protection against pathogens. However, a new role for cells of the immune system has been posited by Ziv and Schwartz who have shown that activated leukocytes play a vital role in maintaining the health of the brain (Schwartz and Ziv, 2008b). The “type of immune activation critically determines the outcome” (Schwartz and Shechter, 2010a, p. 343). The emerging view is that white blood cells, if activated along non-inflammatory pathways, can contribute to brain health and result in behavioral resilience in the face of external stressors. Growth factors, such as BDNF, are featured in the health-promoting activation research as well. In the literature on brain health, there has also been a focus on systemic immunological factors that influence the behavior of brain leukocytes. After examining specifics regarding the state of brain inflammation, a discussion of how the immune system can support brain health and behavioral resilience is presented.

### The inflammatory pathway

Microglia are resident white blood cells in brain. They are essentially the brain’s resident macrophages, which can engulf and destroy pathogens. When microglia detect LPS or the animal is stressed, the microglia become activated and can release the inflammatory cytokines: IL-1, TNF-α, and IL-6 (Monje et al., 2003; Perry et al., 2003; Blandino et al., 2006; Schwartz and Ziv, 2008b; Ekdahl et al., 2009; Molina-Holgado and Molina-Holgado, 2010; Tynan et al., 2010). This is effectively an inflammatory pathway. Blocking microglia’s inflammatory response subsequent to LPS administration will abrogate the emergence of depressed behaviors. Henry et al. (2008) showed that if a drug (minocycline), which prevents activation of microglia, is given prior to LPS administration, then depressive behavior (decreased preference for sucrose) is precluded. Moreover, in the Henry et al. study, minocycline prevented the rise in inflammatory cytokines (IL-1β and IL-6) in the hippocampus as well as attenuating the increased expression of IDO (which is discussed the next section).

Subsequent research has offered clues into phenomenon in brain that occur in response to inflammatory cytokines such as IL-1β that might constitute more proximal causes for changes in behavior. Koo and Duman (2008) showed that pretreatment with an IL-1β inhibitor will block the emergence of depressive behaviors and prevent the decrement in neurogenesis in response to stress. (The significance of decrements in neurogenesis in mediating depressive behaviors will be discussed in a later section.) Downstream of IL-1β is activation of the transcription factor, NF-κB. The changes in behavior induced by IL-1β can be abrogated by blocking the activity of the transcription factor NF-κB, whose activity is increased by IL-1β (Nadjar et al., 2005; Koo et al., 2010). NF-κB serves to further enhance inflammation.

#### The indolamine 2,3 dioxygenase connection

Indolamnine 2,3 dioxygenase has been a focus of much discussion in the literature on inflammation mediating depressive behavior. This enzyme is present in macrophages, as well as other cell types, and operates when a macrophage is activated. It is also active in brain under conditions of chronic inflammation. IDO converts tryptophan to kynurenine and thereby depletes the local environment of tryptophan. Tryptophan is the substrate for the production of serotonin (Miller, 2009).

Some have suggested a causal role for the IDO enzyme in the depressive behaviors associated with inflammation (Miller, 2009; Raison et al., 2010b). The strongest evidence for IDO playing a causal role in LPS or bacille Calmette–Guréin induced depression is the finding that inflammation fails to induce depressed behaviors in mice lacking the indolamine 2,3 dixoxygenase gene or when an inhibitor of the enzyme is given (O’Connor et al., 2009a,b; Raison et al., 2010b). Interestingly, inhibiting the IDO did not decrease the rise in inflammatory cytokines in response to vaccination (O’Connor et al., 2009a). The product of IDO is kynurenine. Systemic administration of kynurenine will induce depression (O’Connor et al., 2009b) and kynurenine can pass through blood brain barrier. In terms of potential ways that kynurenine might impact neurotransmission, kynurenine does get converted to quinolinic acid and kynurenic acid, both of which can affect neurotransmitters signaling such as glutamate signaling and dopamine signaling (Amori et al., 2009; Miller, 2009; Sublette et al., 2011).

Another hypothesis links IDO activity to lower levels of serotonin. Given IDO activity, tryptophan, the precursor to serotonin gets depleted in plasma. Low serotonin has been suggested to play a role in causing depressed behavior (Gal and Sherman, 1980; Miller, 2009; O’Connor et al., 2009a). Against the hypothesis that IDO influences depressive behaviors by lowering tryptophan levels are the findings that tryptophan depletion does not reliably induce depression in all people, although those at risk for depression may be more susceptible (Bell et al., 2001). Moreover, as previously mentioned, kynurenine alone, without any reduction in tryptophan, can induce depression (O’Connor et al., 2009b).

Another puzzling finding is that activation of the IDO enzyme in macrophages in the periphery induces differentiation of T regulatory cells (Henry et al., 2008; Johnson et al., 2009), which as discussed previously, are believed to counter depressive behaviors. At this point, the role of IDO in producing depression given systemic inflammation awaits clarification.

#### The significance of neurogenesis and BDNF

In the literature on neuroprotection, the state of the microglia and whether microglia foster or dampen the release of BDNF has received a great deal of attention. In fact, administration of IL-1β, an inflammatory cytokine, into the hippocampus will decrease the expression of BDNF (Barrientos et al., 2004). The role of BDNF and neurogenesis in depression has also been a focus of attention in the animal literature on depression. The hypotheses that loss of BDNF and downstream diminution of brain growth manifests as depression and conversely that increasing BDNF prevents depression have been investigated. The findings suggest a more complex reality than represented in the initial hypotheses.

Depressed animals and animals subjected to stress do exhibit low levels of BDNF in the hippocampus. Animals subjected to stress also exhibit less neurogenesis in the dentate gyrus of the hippocampus (Lucassen et al., 2011). BDNF induces the growth of dendrites from neurons so that they can make new connections with other neurons (Lucassen et al., 2011). While BDNF is not necessary for neurogenesis, BDNF increases the survival of new cells (Sairanen et al., 2005). Consistent with the possibility that BDNF is diminished in the hippocampus of depressed individuals, depressed individuals have lower hippocampal volume (Bremner et al., 2000; Videbech and Ravnkilde, 2004). Moreover, placing BDNF into the hippocampus (Shirayama et al., 2002), or intracerebroventricular administration of IGF-1, which increases BDNF levels (Carro et al., 2000; Cotman and Berchtold, 2002), exert antidepressant effects in animals (Hoshaw et al., 2005).

Antidepressant drugs do increase neurogenesis in the dentate gyrus of the hippocampus (Santarelli et al., 2003), although this may be limited to adult males (Hodes et al., 2009). Research asking whether the efficacy of antidepressant drugs requires an animal with the capacity for generating new neurons in the dentate gyrus of the hippocampus has yielded inconsistent findings. Irradiation of the subgenual zone of the dentate nucleus of the hippocampus, blocked the efficacy of antidepressants in precluding the behavioral effects of stress in one rat strain (Santarelli et al., 2003). Consistent with this, Airan et al. (2007) found the irradiation of the dentate gyrus precluded the efficacy of antidepressants both on behavioral measures of depression and in increasing neuronal signaling output from the dentate gyrus. However, subsequent work in another rat species did not find that irradiation abrogated the efficacy of the antidepressant fluoxetine in exerting an antidepressant effect nor did those animals displaying lower levels of depressed behavior exhibit increased neurogenesis in the dentate gyrus (Holick et al., 2008). Moreover, some drugs (for example antagonists to melanin concentrating hormone) decrease depressed behavior without influencing neurogenesis (Sahay and Hen, 2007).

Others have looked at whether growth factors are required for antidepressant efficacy. Monteggia et al. (2004) examined the efficacy of antidepressants in blocking the emergence of depressed behavior after exposure to stress. Without an intact gene for expressing BDNF, antidepressants exhibited no efficacy in abrogating the impact of chronic stress (Monteggia et al., 2004).

Nestler’s group has identified the mechanism through which stress alters the expression of BDNF and the mechanism through which antidepressants increase the expression of BDNF. When an animal is subjected to a stressor such as uncontrollable foot-shock or restraint stress, the BDNF gene is harder to express. Two methyl groups are added to the histone 3 at lysine 27 in the promoter region of the gene for BDNF making it difficult to express the BDNF. Antidepressants do not reverse the effects of chronic stress on the BDNF gene; rather, they affect BDNF expression through another mechanism. Nestler has shown that antidepressants decrease the expression of histone deacetylase 5 (HDAC5) in the hippocampus in stressed mice. Histone deacetylases remove acetyl groups from promoter sites so that a gene is less likely to be used to transcribe mRNA for producing a protein (Tsankova et al., 2006; Krishnan and Nestler, 2008). With a lesser amount of a particular HDAC5 present in the hippocampus, the expression of BDNF is increased (Tsankova et al., 2006). However, the story on HDAC5 is complex. Mice with global reductions in HDAC5 are more vulnerable to social defeat and the impact of antidepressants on HDAC5 levels is region specific. Contrary to decreasing HDAC5 expression in the hippocampus, imipramine will increase HDAC5 expression in the Nucleus Accumbens (Tsankova et al., 2006; Krishnan and Nestler, 2008).

At least in some species, antidepressant drug efficacy seems to require growth factors and the capacity for neurogenesis. The previous findings raise the possibility that a lack of BDNF or a lack of neurogenesis in the hippocampus might cause depression. Researchers have investigated this hypothesis. To investigate these questions researchers have used conditional knock outs. Using a conditional knock outs, researchers can control the timing for the loss of the capacity to express a particular protein. Through the use of conditional knock outs, researchers know they are looking at the impact of the loss of functional presence of the protein product of the gene rather than the impact of the loss of the protein during development.

Monteggia et al. (2007) created a conditional knock out mouse for BDNF. Male BDNF knock outs were more hyperactive. Female BDNF knockouts displayed deficits on a forced swim task considered to be a measure of depression and deficit sucrose preference, a measure of anhedonia. Thus, in the Monteggia et al. study, loss of BDNF in increasing depressive behavior is dependent upon gender. Consistent with Monteggia et al.’s male knockouts, Ito et al. (2011) found that their conditional knockout of BDNF in the hippocampus, displayed increased levels of aggression toward conspecifics and did not differ from controls on the forced swim test, a commonly used measure of depression. Thus, the conditional knock out studies fail to support the hypothesis that loss of BDNF in male animals results in behavioral manifestations of depression.

Other researchers have investigated whether blocking neurogenesis induces depression. Irradiating the hippocampus to block neurogenesis does not increase the animals level of depression as evidence by longer latency to feed in a novel situation or immobility on a forced swim test (Santarelli et al., 2003; Airan et al., 2007), nor does blocking neurogenesis in the hippocampus increase the animal’s level of distress as evidenced by failure to groom after uncontrollable stress (Santarelli et al., 2003).

While impaired capacity of cell proliferation in the hippocampus seems to be ruled out as a cause of depression at least under baseline conditions, problems with cell division elsewhere in the brain might play a causal role in producing behavioral depression. Pharmacological destruction of astrocytes cells in the prefrontal cortex (PFC) will induce depressed behavior in an animal. A similar reduction of astrocytes in the PFC occurs in animals exposed to uncontrollable stress. Astrocyte cell death and behavioral signs of depression co-occur whether the astrocyte cell death results from exposure to uncontrollable stress or to a drug which is specifically toxic to astrocyte cells (Banasr and Duman, 2007). Astrocytes do influence neurons. They are a major source of neurotrophins in brain which seems to be induced by dopamine (Miklic et al., 2004). Moreover, the loss of glial cells (both oligodendrocytes and astrocytes) in the subgenual region of the anterior cingulate, the dorsolateral PFC, the supragenual cingulate cortex, and the orbitofrontal cortex, has emerged as marker for major depression (Ongur et al., 1998; Rajkowska and Miguel-Hidalgo, 2007).

#### Location of BDNF expression determines the impact on behavior

Placing BDNF into the hippocampus of an animal will exert an antidepressant effect on a forced swim test and on escape deficit following exposure to inescapable shock (Shirayama et al., 2002). However, an opposite pattern is found in the Nucleus Accumbens. High levels of expression of BDNF in the Nucleus Accumbens released from neurons projecting from the Ventral Tegmental Area will result in depressed behavior (Eisch et al., 2003). In terms of the function of BDNF in the Nucleus Accumbens, Nestler and Carlezon (2006) postulate that expression of BDNF in the Nucleus Accumbens is required for learning connections between external stimuli and negative or positive valences. Thus, the impact of BDNF on ameliorating depressive behaviors is brain area specific.

#### Summary

Thus far, it has been argued that inflammation in the brain is sufficient to create behavioral depression. Investigators have attempted to identify causal mechanisms through which inflammatory cytokines in brain result in depressed behavior. Diminished neurogenesis and/or reductions in growth factors such as BDNF were raised as possible mediating factors of inflammatory cytokines. Inflammation in brain does suppress neurogenesis and the expression of BDNF in the hippocampus. However, given that blocking neurogenesis in the hippocampus or preventing the expression of BDNF fails to create depression, suggests that lack of capacity for neurogenesis in the hippocampus is not sufficient to cause depressed behavior. However, the possibility that inflammation creates depression by blocking cell division in astrocytes in the PFC remains a possibility.

### The health-promoting pathway

As previously discussed, when microglia are activated along an inflammatory pathway, depression is observed. In addition to activation along an inflammatory pathway, microglia can also become activated along several other pathways as well (Michelucci et al., 2009; Derecki et al., 2011). Microglia can assume a form of activation through which they maintain the health of neurons (Butovsky et al., 2006; Schwartz and Ziv, 2008b; Ekdahl et al., 2009; Kosloski et al., 2010). When in the growth promoting mode, microglia release Insulin-like growth factor-1 (IGF-1; Butovsky et al., 2005; Ekdahl et al., 2009; Kosloski et al., 2010; Schwartz and Shechter, 2010a; Ron-Harel et al., 2011). IGF-1 slows inflammation and decreases production of inflammatory cytokines (Pons and Torres-Aleman, 2000; Venters et al., 2001; Park et al., 2011). In addition to being anti-inflammatory, IGF-1 will induce neurons to release BDNF (Carro et al., 2000; Cotman and Berchtold, 2002).

Activation of microglia along the inflammatory pathway versus the growth promoting pathway are mutually inhibitory. TNF-α, an inflammatory cytokine, is inversely correlated the release of IGF-1 from microglia (Butovsky et al., 2006; Schwartz and Shechter, 2010b). IGF-1 can decrease NF-κB transcriptional activity in astrocytes and in TNF-α exposed cells (Pons and Torres-Aleman, 2000). If exogenous IGF-1 is injected into the lateral ventricle along with LPS, then there is less transcription of the genes associated with an array of inflammatory factors including TNF-α, IL-1β, and inducible nitric oxide synthase (iNOS; Park et al., 2011). Dantzer et al. (1999) concomitantly, administered LPS and IGF-1 into the intracerebroventricular area. The IGF-1 administration prevented the emergence of sickness behavior, viz., immobility and reduced exploration of a novel environment that otherwise occurs with LPS administration (Dantzer et al., 1999).

Keeping microglia in a health-promoting state is important. Neurons and astrocytes release factors such as fractalkine and Jagged 1 to keep microglia in a quiescent state (Sestan et al., 1999; Elyaman et al., 2007; Michelucci et al., 2009) and fractalkine will downregulate microglia production of IL-1β in response to LPS (Mizuno et al., 2003; Wynne et al., 2010). However, researchers have also focused on the role of T cells in maintain brain health.

#### The interaction between T cells and the microglia

Schwartz and Ziv have focused on the role that T cells play in determining the behavior of microglia. Schwartz and Ziv (2008a) have proposed that self-reactive T cells with receptors (TCRs) targeted toward central nervous system (CNS) proteins are continually playing a surveillance role. When activated in pro-neurogenesis pathways, they maintain the health of the CNS. T cells, probably by influencing microglia, are vital to the brain’s repair after stroke, for precluding a reduction in levels of BDNF following a psychological stressor, for avoiding symptoms of post traumatic stress disorder (PTSD) after psychological stress, for spatial learning, for neurogenesis stimulated by exercise (Cohen et al., 2006; Ziv et al., 2006; Ziv and Schwartz, 2008a,b; Schwartz and Shechter, 2010a) and for capacity to detoxify reactive alpha-oxoaldehydes (Ron-Harel et al., 2011). T cells are recruited subsequent to activity and/or stress (Schwartz et al., 2009) to the choroid plexus, the meninges, and the Virchow–Robin spaces from which they are able to influence the function of the microglia (Schwartz and Ziv, 2008b; Derecki et al., 2010; Schwartz and Shechter, 2010a,b). The presence of T cells is required for the efficacy of environmental manipulations, such as an enriched environment, to increase the number of microglia releasing IGF-1 in the hippocampus (Ziv et al., 2006; Ziv and Schwartz, 2008a,b; Schwartz and Shechter, 2010a,b). T cells must also be present to witness an increase in IGF-1 in brain given physical activity (Schwartz and Shechter, 2010a).

Support for the importance of T cells in maintaining brain health comes from studies in which all T cells or T cells with TCRs specific for myelin basic protein have been eliminated. Mice that are genetically engineered to lack T cells display severe inability to learn. Replenishing T cells from normal mice into mice which lack T cells can restore the ability to learn and reverse memory deficits (Yirmiya and Goshen, 2011). The importance of T cells has also been demonstrated for the capacity for resilience in the face of stress. Given a stressor such as a predator odor, the expression of ICAM-1 on endothelial cells lining blood vessels in the hippocampus and epithelial cells in the choroid plexus increases to facilitate and guide the migration of T cells, specific for brain proteins, into the brain (Lewitus et al., 2008). When this fails to occur because T cells specific for brain protein have been depleted, more behavioral signs of distress are observed subsequent to stressor exposure (Cohen et al., 2006).

Support for the importance of T cells specific to brain proteins also comes from vaccination studies. Lewitus et al. (2008, 2009) vaccinated animals with a protein found in the CNS, myelin basic protein, but which had been altered to make the myelin basic protein less likely to induce a strong inflammatory response. Then the animals were subjected to stressful conditions, which had been previously shown to induce depressive behavior as well as decreasing the level of BDNF in the hippocampus. After exposure to the stressful conditions, the animals that had been vaccinated failed to display decreased sucrose preference, a measure of anhedonia. Moreover, the level of BDNF in the hippocampus remained at control levels. Consistent with higher levels of BDNF, the immunized animals exhibited more neurogenesis in the hippocampus.

#### Which type of T cell?

In the Immunology literature, the identification of the various paths along which T cells can be differentiated has been a vigorous area of investigation. Five major types of T cells have been identified: Th1, T follicular helper cells, Th2, Th17, and Tregs. Two types are germane here: Th2, which release IL-4, IL-3 and IL-13, and Tregs (Mimran and Cohen, 2005; Murphy, 2012).

In the literature on the importance of T cells for the health of the CNS, the topic of which type of T cells exert a salubrious effect has not been a major focus of attention. However, given the various roles that cells of the immune system can play in the brain raises the issue of which particular cytokines and which particular type of T cells promote brain health. Yirmiya and Goshen, commenting on research by Derecki and others (Derecki et al., 2010; Yirmiya and Goshen, 2011) noted that T cells present in meninges secreting IL-4 were important for the capacity for learning. Presumably, these cells were Th2 differentiated a non-inflammatory pathway. Butovsky et al. (2006) showed that IL-4 induced microglia to release IGF-1.

Some studies have asked about the impact of Tregs in influencing brain inflammation. Findings on the impact of Tregs have been puzzling (Walsh and Kipnis, 2011). Kipnis et al. (2002) found that Tregs, in the choroid plexus and meninges of the brain, dampen the activity of other T cells so that less repair after injury occurs. When Tregs are depleted, the animal exhibits stronger recovery after crush injury of the optic nerve. Ziv et al. (2007) also found that for promoting neurogenesis in brain after stroke, downregulating Tregs improves outcome. Moreover, subsequent work (Cohen et al., 2006) showed that depleting an animal of all Tregs while maintaining T cells specific for myelin basic protein precluded signs of distress following exposure to the odor of a predator. In some strains, the T effector cells and Tregs seem to cancel each other’s protective effects (Kipnis et al., 2004).

There are other studies that suggest a salubrious role for T cells in promoting brain health. Reynolds et al. (2007, 2009a,b) have shown that co-culturing microglia with Tregs will attenuate reactive oxidative species (ROS) and NF-κB transcriptional activity subsequent to antigen exposure. Employing vaccination as a mechanism for increasing a T regulatory response has also been shown to yield neuroprotection. Researchers have vaccinated animals with vasoactive intestinal peptide. Vasoactive intestinal peptide elicits the differentiation of T cells into Tregs. Subsequently the animals were exposed to 1-methyl1-4-phenyl-1,2,3,6-tetrahydropyridine (MPTP), a chemical which will cause degeneration of neurons in the substantia nigra, creating symptoms of Parkinson’s disease. The animals that had been vaccinated showed resistance to the development of Parkinson’s disease (Kosloski et al., 2010; Reynolds et al., 2010).

Whereas the previous studies, examined the impact of Tregs, other studies have focused on identifying cytokines that exert a neuroprotective effect. IL-10, a primary cytokine released by Tregs and microglia, blocks neuronal apoptosis after injury (Zhou et al., 2009). Interferon-γ (IFN-γ) and TGF-β also exert an influence of microglia which drives neuroprotection (Kipnis et al., 2004). Warner-Schmidt et al. (2011) argue that IFN-γ exerts an antidepressant effect by increasing levels of p11, a protein involved in trafficking serotonin receptors to the cell membrane. Thus, Warner-Schmidt et al. posited a role for IFN-γ beyond influencing further inflammation. Given the inconsistencies in impact across cytokines, it is important to replicate findings in various species to determine whether findings are species specific.

#### What can be stated with confidence?

The mechanisms through which leukocytes play a role in maintaining the health of the CNS is an area of intense research having implications for degenerative diseases as well as mood disorders. The interaction between T effector cells, Tregs, microglia, and neurons requires more elucidation with recognition that findings may not generalize across species or across strains within a species (Walsh and Kipnis, 2011). The research on whether Tregs are beneficial or not has been particularly inconsistent. How Tregs impact brain inflammation may depend upon which cells the Tregs are influencing. Future work may further identify which types of T cells and which cytokines foster brain health versus inflammatory responses. However, in the human literature, Tregs are prominently featured in work on the Hygiene Hypothesis.

#### The hygiene hypothesis

The hygiene hypothesis attempts to explain why persons in the third world have much lower rates of disorders associated with inflammatory conditions: Parkinson’s disease, depression, anxiety disorders, asthma, and inflammatory bowel disease. According to the hygiene hypothesis, persons in the third world are exposed to more pathogens, such as helminthes (parasitic worms) and pathogens found in human feces, such as *M. vaccae*. These particular pathogens are good at eliciting the differentiation of T cells into Tregs (Rook and Lowry, 2008, 2009; Raison et al., 2010c). (As previously discussed, Tregs will dampen inflammation.) Downregulating inflammation systemically, will preclude inflammatory cytokines in brain, thereby decreasing depression and anxiety.

There are studies on how early life exposure to pathogens impacts resilience or vulnerability to stress. Consistent with the idea that early life exposure to pathogens is protective, Bilbo et al. (2008) found that early life exposure to *E. coli* protected mice from stress. As adults, when the mice were exposed to uncontrollable shock, they displayed less diminution of social exploration and less elevation in cortisol during the stressor. Contrary to the finding that early life exposure to *E. coli* promotes resistance to stress, exposure to particular bacteria for example, type A2/Singapore influenza, may increase risk for later depression (Bale et al., 2010). So does early life exposure to infection promote resilience or vulnerability to stress? A testable hypothesis asserts that when a pathogen elicits Tregs, the pathogen promotes resilience; whereas when the pathogen fails to provoke Tregs, exposure promotes vulnerability. As findings accumulate identifying pathogens which are powerful in eliciting Tregs, some of the discrepancies in the literature may be resolved.

Lowry has focused on those particular neuronal circuits that are activated when a T regulatory response occurs. Subsequent to eliciting T regulatory response through vaccination with attenuated *M. vaccae*, serotonergic neurons in the interfascicular dorsal raphe were activated (Lowry et al., 2007). This resulted in a faster latency to swim on the forced swim test, a laboratory measure of resilience to depression. Inoculation with bacteria that failed to elicit a T regulatory response did not have this effect. Elsewhere, Hale and Lowry (2011) have differentiated the various serotonergic circuits in the raphe. Some of these serotonergic neurons (in the caudal, dorsal raphe) are involved in creating anxiety and learned helplessness (Maier et al., 2006). The interfascicular dorsal raphe neurons project to the medial PFC and are part of a feedback loop that will downregulate the caudal dorsal raphe serotonergic neurons to decrease learned helplessness. In terms of a mechanism for the antidepressant effect of vaccination with *M. vaccae*, the *M. vaccae* activate Tregs which activate the interfascicular dorsal raphe neurons that suppress the anxiogenic/depressogenic circuitry (Lowry et al., 2007, 2008). Lowry et al.’s research is important because it raises the possibility that Tregs not only confer resilience to stress by countering depressogenic inflammatory activation, but also may confer resistance through a direct impact on neuronal function. Lowry’s research suggests that Treg cytokines can induce activity in interfascicular dorsal raphe neurons. The interfascicular dorsal raphe neurons inhibit learned helpless behaviors.

### Summary

The case has been advanced that depression reflects an inflammatory state in the brain. An inflammatory state in the brain can be induced by inflammatory cytokines in the periphery or by psychological stressors. While some have focused on how brain inflammation results in manifestations of depression, others have examined resilience. Microglia are the source of inflammatory cytokines, associated with depressive behaviors, as well as the source of IGF-1, associated with resilience. Events downstream of microglia behavior have been investigated. Researchers have attempted to identify which consequences of inflammatory cytokines result in depressive behaviors. Inflammatory cytokines do decrease the expression of growth factors (e.g., BDNF), which in turn impact neurogenesis. However, findings fail to support the view that a simple lack of BDNF or an incapacity for neurogenesis in the hippocampus redounds in depression. Moreover, the impact of BDNF varies according to the structure in the brain under observations. Attempts to link cytokine levels in brain to a particular neurotransmitter’s function to explain depressive behaviors or resilience have been made. Lowry’s work implied Treg anti-inflammatory cytokines influence particular serotonergic neurons to yield stress resilience. In fact, cytokines impact other neurotransmitter systems as well (Pace et al., 2012). Thus, how cytokines impact neurotransmitters awaits further elucidation. The work on how microglia can protect the brain and confer behavioral resilience to stress has focused on identifying determinants of microglia behavior, that is whether the microglia release inflammatory cytokines or IGF-1. Questions regarding which particular cytokines, emanating from specific T cell types, are most impactful in inducing the microglia to be in a health and resilience promoting state have been asked. Studies examining the role of Tregs has yielded some contradictory findings. The future promises a more articulated story on how the behavior of microglia, which is influenced by specific types of T cells and the particular cytokines they release, can result in depressive behavior or behavioral resilience.

Although the research does not yet offer an articulated mechanism for the ways in which inflammation mediates depressive mood and behaviors and conversely how healthy microglia confer resilience, it does appear that systemic inflammation is an influential link in the causal chain of depression. This, in turn, suggests that we would be well advised to consider a variety of ways to reduce systemic inflammation. The ways in which antidepressants impact the immune system are now considered.

## Impact of Antidepressant Drugs on Inflammation

Antidepressant drugs have become the standard treatment for people suffering from depression, often being used even when the patient is receiving psychological treatment. Given that inflammation is involved in depression, possibly causally, it is useful to examine the effects of antidepressant drugs on inflammatory state, and also on whether the drugs can counteract conditions leading to inflammation. Most of the studies that have been done on short term influences of the drugs, in both animals and people. However, it has become fairly standard for patients to remain on antidepressants for years at a time. Given that these drugs have deleterious side effects, it would be important to continue using them only as long as they are effective. Therefore we examine the effects of antidepressants on inflammation in two sections: one on short term use, and a separate one to see what can be discerned from studies of people who have taken antidepressants for years.

### Short term studies

In the human literature, the efficacy of antidepressants has been challenged (Kirsch, 2010). However, there is support for efficacy in the animal literature. One causal factor in systemic inflammation is stress. Studies have examined whether antidepressants can abrogate the impact of stress. When animals are subjected to uncontrollable stress, the extent of their distress can be gaged by the reduction in their preference for sucrose consumption. Also, they will fail to escape from an aversive stimulus. It turns out that both of these stress responses can be abrogated by antidepressant treatment (Bessa et al., 2009). Another way to produce stress in animals is to put them in a novel environment. After exposure to a novel environment, stress is manifested on a number of measures. Treatment with antidepressants will decrease immobility in a forced swim test, will increase exploration in a novel environment, and will preclude the decrement in food consumption produced by a novel environment (Dulawa et al., 2004).

Questions about how selective serotonin reuptake inhibitors (SSRIs) achieve their efficacy have been raised. Maes and colleagues, using human subjects, have shown that if SSRIs are mixed with whole blood outside of the body, the production of anti-inflammatory cytokines is increased while the production of proinflammatory cytokines is decreased (Maes et al., 1999; Kubera et al., 2001a). Obuchowicz et al. (2006) also showed in cultured glial cells taken from animals, antidepressants will dampen the release of inflammatory cytokines in response to LPS. In other studies, patients who were treated with antidepressants for a short period of time have undergone blood draws and then cytokines have been measured. Those persons taking antidepressants for 5–8 weeks do exhibit lower levels of IL-6 levels in plasma (Sluzewska et al., 1995; Basterzi et al., 2005); lower TNF-α levels (Lanquillon et al., 2000; Tuglu et al., 2003); lower levels of monocytes and neutrophils in plasma (Seidel et al., 1996; Tuglu et al., 2003); higher levels of IL-10 (Kubera et al., 2001b); and higher levels of Tregs (Himmerich et al., 2010). Moreover, when whole blood from persons taking antidepressants for 6 weeks are tested in response to a mitogen stimulus, the ratio of IFN-γ/IL-10 is lower (Maes et al., 1999; Kubera et al., 2001a). Thus, the case can be made that antidepressants, taken for several weeks, exert anti-inflammatory effects as seen by specific changes cytokines, and thus offer a likely explanation for the initial positive effects ascribed to antidepressant drug use.

### Long-term studies

There have been studies examining the long-term impact of antidepressants on systemic inflammation. Many of these studies have examined the impact of antidepressants on phenomena that are associated with increased systemic inflammation: diminished heart rate variability, weight gain, metabolic syndrome, type II diabetes, and atherosclerosis. These studies as well as the studies examining the impact of long-term antidepressant use on the inflammatory marker of CRP are reviewed.

#### C-Reactive protein

C-Reactive protein is an acute phase protein released by the liver in response to IL-6. It is considered to be a marker for systemic inflammation (Blake and Ridker, 2002; Pearson et al., 2003). Dawood et al. (2007) noted that treatment with an SSRI for 12 weeks significantly increased high sensitivity CRP. This finding was consistent with Hamer et al.’s (2011) findings from two data sets. In a data set of 4,584 research-participants from the Whitehall studies of British civil servants, long-term use (10 years) of an antidepressant was associated with higher CRP after controlling for psychological distress. In a large sample of 8,131 Scottish research-participants from another data set, an association was noted between higher CRP levels and treatment with tricyclic antidepressants (TCAs) although no association was noted for those treated with SSRIs. The duration of medication use and dosage were not reported in the Scottish sample. However, Hamer et al. noted that in the Whitehall study antidepressant use at only one data point was not associated with CRP elevations. Thus, short exposure to antidepressants probably is insufficient to raise CRP levels.

#### Heart rate variability

Both the sympathetic and parasympathetic nervous systems innervate lymph nodes, as well as the heart. In terms of innervation of the heart, more parasympathetic control over the heart rate is manifested as greater heart rate variability (Porges, 2007, 2009). Greater heart rate variability is considered to reflect better coordination between breathing and heart function. Moreover, decreased heart rate variability is a predictor of heart attacks and cardiovascular disease (Hayano et al., 1990, 1996). In terms of innervation of lymph nodes, acetylcholine from the post ganglion neuron of the parasympathetic nervous system will inhibit release of TNF-α, IL-1, IL-6, and IL-18 from macrophages. In the liver, greater vagal tone will inhibit NF-κB activation and thus the release of acute phase proteins (Pavlov and Tracey, 2005; Rosas-Ballina and Tracey, 2009). Thus, increasing vagal tone can be expected to decrease systemic inflammation.

Several meta-analyses and studies of non-medically ill patients treated with tricyclics for short periods of 6–16 weeks, have noted a decrease in heart rate variability (Tulen et al., 1996; van Zyl et al., 2008; Kemp et al., 2010). Results for SSRIs taken over a short period of time have been inconsistent with several small N studies noting a decrease in heart rate variability (Dawood et al., 2007) and others finding no impact (Davidson et al., 2005; van Zyl et al., 2008; Kemp et al., 2010).

The question of whether treatment with antidepressants makes long-term outcomes less favorable requires investigation of how antidepressants impact HRV when taken for over a year. In contrast to studies assessing short term impact of treatment, a study by Licht et al. (2008) examined the impact of antidepressants on persons with either remitted or current depression who were followed for 8 years. Two measures of heart rate variability were employed in the study: standard deviation of normal-to-normal (SDNN) beats and respiratory sinus arrhythmias (RSA). The study included 67 persons taking TCAs, 435 persons taking SSRIs, and 137 taking other antidepressants. There were 1,018 persons who were depressed or had been depressed but were not taking medications. The study also included 524 controls who had never been depressed. Thus, there was a control group of persons who were never depressed as well as persons who had been or were currently depressed broken down according to medication status. The results were that those taking medications exhibited lower heart rate variability when compared to never-depressed controls (for SDNN, *d* = 0.207–0.849; for RSA, *d* = 0.413–0.862 across various medications). Comparing those remitted depressed not on medication or still depressed not taking medication did not differ from controls on SDNN beats and did not differ as greatly from controls on RSA (*d* = 0.118). Licht et al. noted a dose–response relationship between antidepressants and reduced HRV. Licht et al. concluded that the association between lowered HRV and depression (p. 1358) “appears to be mainly driven by the effect of antidepressants.”

#### Weight gain

Obesity is considered to be an inflammatory state. Adipocytes are capable of releasing IL-6 and TNF-α. It is estimated that 30% of the IL-6 in circulation derives from adipose tissue. Moreover, omental fat tissue (fat around the abdomen) is populated by activated macrophages releasing IL-6 (Mohamed-Ali et al., 1997; Hamer, 2007). Even in normal weight individuals, omental fat can be inflammatory (Shelton and Miller, 2010). Weight reduction can be expected to decrease systemic inflammation.

A meta-analysis of the impact of antidepressants on weight gain suggested a strong effect for the TCAs and an effect for some SSRIs (paroxetine) in studies examining long-term exposure to antidepressants (over 6 months; Serretti and Mandelli, 2010). The meta-analysis was consistent with an early review by Fava (2000) and a review by Deshmukh and Franco (2003) which concluded that TCAs and paroxetine were particularly likely to be associated with weight gain. A study by Kivimaki et al. (2010) found a correlation between duration of treatment with antidepressants, both TCAs and SSRIs, and greater weight gain. Moreover, a study of persons treated with antidepressants for panic disorder for a year, noted significant weight gain for all of the SSRIs (citalopram, fluoxetine, fluvoxamine, and paroxetine) used in the study (Dannon et al., 2007).

#### Metabolic syndrome

As noted above, weight around the abdomen is inflammatory. Weight around the abdomen is a component of metabolic syndrome. In a cross-sectional, large *N* study (25,315 subjects), Raeder et al. (2006) found an association between abdominal obesity, general obesity, higher cholesterol levels (components of the metabolic syndrome), and the use of SSRIs after controlling for symptoms of anxiety and depression. Associations were particularly pronounced among persons in their 40s who were taking paroxetine and were absent in those taking citalopram. The authors did not have data on duration of exposure, however, they remarked that they expected metabolic syndrome to take some time to develop.

#### Diabetes

Inflammatory cytokines create insulin resistance. Type II diabetes, characterized by insulin resistance, is an inflammatory disease (Dandona et al., 2011). Thus, an increase in type II diabetes might be considered a proxy for an increase in systemic inflammation.

In a case-controlled study, persons who were all depressed at entry into the study were followed for 2.8 years. Of those entering the study, 2,243 persons developed diabetes during the follow-up period. Those who developed diabetes were matched on age, gender, and time in the study with 8,963 comparison subjects, who had not developed diabetes. A comparison of long-term antidepressant users versus short term users yielded no difference on their initial severity of depression. However, those developing diabetes were more likely to have been treated with antidepressants at moderate to high doses for over 2 years prior to the onset of diabetes. The increased risks obtained for amitriptyline, fluvoxamine, paroxetine, and venlafaxine (Andersohn et al., 2009).

Several other large-sample-size-studies have yielded findings consistent with the hypothesis that long duration of exposure antidepressants increase the risk of Type II diabetes. In a prospective study of 9,197 participants followed for 4.8 years, treatment duration of over 6 months of an antidepressants (both the TCAs and the SSRIs), doubled the risk of Type II diabetes. Duration of exposure to the antidepressant was associated with elevated risk for diabetes in both those with mild and moderate depression (Kivimaki et al., 2010). In a third large *N* study (3,234 persons with risk factors for diabetes), with a follow-up of 10 years, Rubin et al. (2010) also found that continuous use of antidepressant use increased the probability of developing diabetes. In those treated with metformin, antidepressant use did not increase the risk of diabetes development. The previously discussed study by Raeder et al. (2006) also found that paroxetine was associated with higher rates of diabetes.

#### Atherosclerosis

There is an association between major depression and heart disease (Frasure-Smith and Lespérance, 2006). Atherosclerosis, like depression, is an inflammatory disease and inflammation contributes to the calcification of the aorta (Kritharides et al., 2000). In a study of heart patients after controlling for depressive symptoms, antidepressant use was found to be associated with greater aorta calcification (Shah, 2011).

## Long-Term Effects of Antidepressants: A Mechanism for Creating a Chronic Course

Previous sections have advanced the case for depression as an inflammatory condition, with strong evidence for the conclusion that inflammation is an important link in the cause of depression. The evidence in this section points to the conclusion that short term use of current antidepressant medications reduces inflammation, and ameliorates depression. But additional data strongly suggest that the positive effects are reversed with prolonged use. That is, it is not merely the case that antidepressant medications appear to lose their efficacy with prolonged use, but rather, they promote reverse effects, increasing inflammation.

Contrasting data on the probability and frequency of depressive relapses prior to the advent of pharmacological treatments to the era after, Fava and Offidani (2011) along with an earlier paper by Fava (2003) raised the possibility that antidepressants create a chronic course in major depression. Littrell (1994) also contrasted the duration of wellness intervals between episodes of depression in persons before and after the advent of modern medications and also noted that antidepressants shorten the duration of wellness intervals. Fava and Offidani (2011) considered various possible explanations for the mechanism through which antidepressants contribute to chronicity of depression including tolerance, lowering the brain’s threshold for mood fluctuations, and withdrawal phenomenon from antidepressants. Another possibility is that antidepressants, when taken for over a period of 6 months, increase systemic inflammation such that depression becomes chronic. Evidence suggests that when taken over years, antidepressants increase systemic inflammation. Although increased inflammation fails to obtain for particular classes of antidepressants (SSRIs versus TCAs) on some measures, both classes of drug have been associated with inflammation on at least one index. Thus, there is evidence of increased inflammation for both classes of antidepressants.

The previously reviewed literature suggests that sustained exposure to antidepressants for over a year is associated with more systemic inflammation. The mechanisms through which long-term use antidepressants might increase systemic inflammation have not been investigated. Identifying these mechanisms is important for future research. The other lesson emerging from this literature is that evaluating drugs employing the usual 8 weeks of assessment customary in drug trials is not sufficient for determining the efficacy of pharmacological interventions. When the assumption is that an individual will be exposed to a chemical over an extended period, years rather than weeks are required.

### Way forward

Research implicates inflammation as a proximal causal factor in producing depressive symptoms. At present, psychological treatments for depression such as interpersonal therapy and Cognitive Behavior Therapy have not been evaluated for their impact on inflammation. In evaluating interventions, psychologists may wish to include measures of inflammation, such as CRP, as dependent measures. If psychological interventions decrease systemic inflammation, then there would be evidence that psychological treatments not only decrease levels of depression through a direct pathway but also through the indirect pathway of impact on systemic inflammation.

Systemic inflammation does contribute to depression. Over the long run, antidepressants contribute to inflammation. If antidepressants either lose efficacy or contribute to inflammation in the long run, other mechanisms for decreasing inflammation must be relied upon for treating depression. The range of strategies for improving mood is broadened by this research. Consumption of omega-3 fatty acids (Kiecolt-Glaser, 2010), adherence to a Mediterranean diet (Dai et al., 2008; Sanchez-Villegas et al., 2009), and consumption of curcumin (Aggarwal, 2011) lower inflammation and lower the risk for depression. In animal work, curcumin has been shown to exert antidepressant effects of similar magnitude to antidepressant drugs (Wang et al., 2008). Interventions such as exercise (Hamer, 2007), yoga (Bernardi et al., 2001; Kiecolt-Glaser et al., 2010), meditation (Pace et al., 2009) have been shown to dampen inflammation. Meditation (Pace et al., 2009), exercise (Greenwood and Fleshner, 2008, 2011), and consumption of omega-3s (Kiecolt-Glaser et al., 2011) have been demonstrated to confer resilience to stress. Psychologists have a long tradition in strengthening behavioral compliance and have the skills for assisting individuals in adhering to life style changes. The recognition that depression is an inflammatory disease ushers in a wealth of new possibilities for treating and preventing mood disorders.

## Conflict of Interest Statement

The author declares that the research was conducted in the absence of any commercial or financial relationships that could be construed as a potential conflict of interest.
